# Mechanisms of plant–insect interaction

**DOI:** 10.1093/jxb/eru503

**Published:** 2015-01-07

**Authors:** Robert D. Hancock, Saskia Hogenhout, Christine H. Foyer

**Affiliations:** The James Hutton Institute, UK; John Innes Centre, UK; University of Leeds, UK

The study of plant–insect interactions continues to be an exciting and fast-moving field that builds upon the more extensive literature available in plant–microbe interactions and offers new and significant insights into both the unique molecular determinants of plant–insect interactions and the wider ecological context. This special issue of the *Journal of Experimental Botany* brings together a collection of reviews and original research papers that reflect the breadth and quality of work undertaken in contemporary research into plant–insect interactions.

The review by Mark Mescher and Consuelo De Moraes (2015) gives a historical perspective of the often-controversial field of plant sensory perception and how this relates to the capacity of plants to recognize and respond to the presence of both beneficial and detrimental insects. These authors provide an overview of the acute sensory capacity of plants, revealing the emerging story of mechano- and chemoreception. The exquisite capacity of plants to recognize subtle differences in mechanical stimuli is highlighted, with the suggestion that this is crucial in distinguishing mechanical stimuli caused by insects from those caused by other external factors such as wind and rain. These capabilities are supplemented with a highly evolved capacity for chemoreception that not only allows plants to respond to direct insect attack but also provides a mechanism for recognizing chemical elicitors associated with processes such as egg laying, allowing anticipatory upregulation of defensive traits prior to larval hatching. Combined with a discussion of the capacity for detection and response to insect-induced volatile emissions from neighbouring plants, this review highlights the sophistication and complexity of plant sensory responses to an ever-changing external environment. A parallel and complementary theme is explored in the review by Simon Zebelo and Massimo Maffei (2015), which focuses on the early signalling events following insect infestation in plants. Highlights include recent findings regarding the role of electrical signalling in plant–insect interactions and the integration of early and late signalling events that are mediated via hormonal signals among other things. Joe Louis and Jyoti Shah (2015) continue the theme of signalling in plant defence with their review on the role of PAD4 in aphid resistance. This protein exhibits key functions in both basal and effector-triggered immunity against microbial pathogens and is also an integral component of salicylic acid (SA) signalling and response. However, as Louis and Shah argue, it has distinct molecular activities in response to aphid infestation that differ from its role in defence against microbes.

Toby Bruce (2015) provides an interesting discussion of the mechanisms of plant recognition of insect attack as well as downstream signalling and defence mechanisms, but broadens the subject by also introducing mechanisms by which insects recognize their hosts and overcome plant defences. A key theme of this review is the significance of the temporal response of both partners in the interaction and role of evolutionary timescales in driving both host and insect differentiation. This review introduces the concept of insect effector proteins that modulate host immune responses in favour of the attacker, an area of considerable research interest at present. Furthermore, the review underlines the importance of the ecological context of the plant–insect interaction and the role that additional organisms interacting with either the host plant or the insect can have on the outcome of the primary interaction. This concept is covered in more detail by Akiko Sugio and colleagues (2015), who specifically focus on the influence of plant- or insect-associated bacteria on the outcomes of plant–insect interactions, an area likely to become more significant in the near future. They highlight published literature that illustrates how pathogenic or beneficial microbes associated with plants can modulate the plant–insect interaction in favour of either of the partners.

Two reviews illustrate the utility of ‘omics’ technologies in dissecting the molecular basis of plant–insect interactions. Pankaj Barah and Atle Bones (2015) provide an overview of a range of ‘omic’ technologies and how these have been applied to this subject area. They highlight the need for integration of data sets obtained from various platforms and discuss the need to study plant–insect interactions within the context of the ecosystem within which they occur. Finally, they discuss mathematical approaches for integrating different data sets and highlight the challenge of modelling complex plant–insect interactions within a realistic ecosystem defined by multiple biotic and abiotic interactions. Foyer *et al.* (2015) conducted a systematic review of previously published microarray data to identify core components of the *Arabidopsis* transcriptional response to phloem-feeding insects. Their analysis shows surprisingly little overlap in terms of the transcripts expressed in different studies in accordance with the central tenet of Toby Bruce’s paper highlighting the dynamic nature of plant–insect interactions. Despite the disparity of responses between different studies, their work highlighted the significance of cell wall damage in the perception of aphid attack, an unexpected finding given the paradigm that phloem-feeding insects have adopted a stealthy mode of feeding that causes minimal damage to plant tissues. Another key finding of this review was the observation that feeding by generalists, but not specialists, resulted in transcriptional inactivation of glucosinolate biosynthesis, supporting the hypothesis that the generalist insect has the capacity to modify the plant immune system via effectors.

Beyond the fascination of the subject of plant–insect interactions, a key motivation for studies is the economic significance of agricultural pests that are estimated to cause worldwide crop losses amounting to hundreds of billions of dollars annually (Kerchev *et al.*, 2012). The review by Marion Harris and colleagues (2015) illustrates both how curiosity-driven fundamental research can provide novel insights to drive methods for crop protection and how breeding for insect-resistant crops can lead to the development of a greater mechanistic understanding of the plant–insect interaction. This is illustrated by a historical discussion of the development of resistance gene theory and how these developments assisted in the accumulation of our present day knowledge concerning effector-triggered immunity. The final review by Furch *et al.* (2015) discusses the role of salivary effectors in plant–aphid interactions. They focus on salivary proteases that have the capacity to degrade sieve element proteins, facilitating feeding by preventing protein-mediated sieve element occlusion. Furthermore they discuss several mechanisms by which the plant may protect its sieve element proteins from degradation, including protein glycosylation and the presence of sieve element-located protease inhibitors.

The original research papers in this volume represent a spectrum of current insights into the molecular mechanisms of plant–insect interactions. As discussed above, one of the most exciting developments in the field is the fast-moving research on insect effectors, which enable insect colonization of plants via modulation of plant processes, including probable defence pathways. The knockdown of effector genes by RNA interference (RNAi) is a key technology for the identification of aphid effectors and subsequent functional characterization of these proteins. Recently, it was demonstrated that aphid gene knockdown is achieved by plant-mediated RNAi in which the dsRNAs are introduced into the aphid by feeding the insects on transgenic plants that transiently or stably produce the dsRNAs. In this volume, a research paper from Saskia Hogenhout’s group (Coleman *et al.*, 2015) presents information regarding the persistence of RNAi following removal of aphids from the dsRNA-producing plants. Significantly, the paper shows for the first time that RNAi is transferred to subsequent aphid generations. The experiments illustrate how plant-mediated RNAi is a useful tool for investigating the contributions of specific aphid effectors in plant colonization over multiple aphid generations.

Fiona Goggin’s laboratory has contributed two papers. Pallipparambil *et al.* (2015) show that the well characterized *Mi-1.2* resistance gene, which provides tomato plants with resistance to a range of phloem-feeding insects and to root-knot nematodes, also has a negative impact on the commonly used biocontrol agent *Orius insidiosus*. This zoophytophagous predator is widely used in integrated pest and disease management (IPDM) to control thrips, mites, and aphids. Whereas *O. insidiosus* mostly feeds on insect eggs, it is known to be omnivorous and also feeds on pollen, xylem sap, and the contents of epidermal and mesophyll cells. The paper by Pallipparambil *et al.* (2015) demonstrates higher total mortality of *O. insidiosus* when reared on moth egg-infested *Mi-1.2* plants than on control plants lacking the *Mi-1.2* resistance gene. They further demonstrate that mortality is primarily associated with early stages of *O. insidiosus* development that are more dependent on plant feeding than the later stages. As *O. insidiosus* is not a known phloem feeder, this suggests that *Mi-1.2*-mediated resistance can occur outside the phloem. *Mi-1.2* expression analyses confirmed expression in the phloem, but also in epidermal and mesophyll cells. The findings illustrate the ecological complexity of plant–insect interactions and their significant implications for IPDM programmes. In Wu *et al.* (2015), the role of *Mi-1.2* is placed within the broader context of tomato signalling and defence responses to aphid infestation. They found that transcripts of the ethylene response factor (ERF) *Pti5* are upregulated in tomato plants colonized by *Macrosiphum euphorbiae*. Suppression of this ERF had a similar positive impact on aphid survival and fecundity irrespective of the genetic background (with or without *Mi-1.2*) of the host plant, suggesting that *Mi-1.2* and *Pti5* activate separate immune pathways. Experiments with various mutant lines revealed that *Pti5* expression was independent of SA, jasmonic acid (JA), and ethylene signalling. However, the interaction between ethylene signalling and *Mi-1.2* is complex, because ethylene promotes both host acceptance and aphid fecundity, but only in a genetic background lacking *Mi-1.2*. These data highlight the complexity of signal transduction pathways and the role of crosstalk in the development of the immune response that was also highlighted in some earlier reviews.

Other original research papers in this volume focus on the roles of secondary metabolites in plant resistance to insects and nematodes. Two papers from Georg Jander’s laboratory combine QTL and phytochemical analyses to examine the roles of secondary metabolites in maize resistance to insects. Betsiashvili *et al.* (2015) analysed two loci on chromosomes 4 and 6 associated with resistance to the aphid *Rhopalosiphum maidis*. The locus on chromosome 4 contained several genes involved in the biosynthesis of the known resistance factor 2,4-dihydroxy-7-methoxy-1,4-benzoxazin-3-one (DIMBOA) and aphid resistance was associated with increased DIMBOA content. DIMBOA has previously been shown to be an essential contributing factor for callose formation in maize. In agreement with this, lines that produce high DIMBOA levels exhibited increased callose formation in response to aphid feeding. However, it was unclear if resistance was a result of direct DIMBOA toxicity or mediated via enhanced callose formation. A QTL on chromosome 6 also contributes to the aphid resistance phenotype, indicating an additional resistance mechanism independent of DIMBOA. Yan *et al.* (2015) presented evidence for the anti-insect effects of the non-protein amino acid 5-hydroxynorvaline in maize. This amino acid was shown to increase in leaves in response to feeding by aphids and caterpillars, and treatments with methyl jasmonate, SA or abscisic acid, but was reduced upon ethylene treatment. In artificial diets, concentrations as low 100µM, similar to the concentration found in maize leaves, inhibited aphid reproduction thereby suggesting that the compound has anti-insect properties. The paper also identified two QTLs that combined accounted for nearly 40% of the phenotypic variation, providing opportunities for rapid introgression of potential resistance into commercial varieties.

Atle Bone’s laboratory has previously used a novel technique to produce oilseed rape plants that lack myrosinase-containing myrosin cells, which are normally distributed throughout plant tissues (Ahuja *et al.*, 2011). In this volume, the paper by Ahuja *et al.* (2015) describes a series of experiments in which *MINELESS* plants were used to dissect the role of glucosinolate breakdown products in plant resistance to the cabbage moth *Mamestra brassicae*. *MINELESS* plants exhibited negligible levels of myrosinase activity and had higher total glucosinolate concentrations in both the absence and the presence of *M. brassicae* larvae with altered abundance of individual glucosinolates. Whereas glucosinolate hydrolysis products were less abundant in *MINELESS* plants than in the wild type, two specific hydrolysis products, 1-methyl thiopentane and 1-methyl thiohexane, were much more abundant in the *MINELESS* plants in both the absence and the presence of *M. brassicae* larvae. *MINELESS* plants also exhibited altered gene expression patterns relative to wild-type plants and exhibited weaker induction of jasmonate synthesis and signalling in response to *M. brassicae* feeding. Behavioural assays indicated that *MINELESS* plants were less attractive to *M. brassicae* larvae and in no-choice experiments, larvae performed less well on the myrosinase-ablated lines. These data indicate the complexity of secondary metabolite defences in which reduced performance is possibly associated with higher levels of intact glucosinolates or higher levels of specific glucosinolate hydrolysis products. The results presented in this paper also indicate a role for glucosinolates or their breakdown products in phagostimulation.

Nematodes contribute to the ecology of plant–insect interactions. Whereas plant-parasitic nematodes attack plants, entomopathogenic nematodes help to defend plants against insect pests and may be recruited to the plant via volatiles that are produced following insect herbivory on roots (Rasmann *et al.*, 2005). Hiltpold *et al.* (2015) examined the impact of root exudates on nematode quiescence and activity. They demonstrate that a heat-stable factor found in pea root exudates has the capacity to induce a quiescent state in both plant-parasitic and entomopathogenic nematodes when supplied at relatively high concentrations. This has practical applications for IPDM, because nematode quality traits were maintained for longer when stored in root exudates than when stored in water. The presence of root exudates at concentrations found in the rhizosphere increased the activity of entomopathogenic nematodes while reducing that of plant-parasitic nematodes. Furthermore, increased entomopathogenic nematode activity was associated with increased nematode-infection rates of *Galleria mellonella* larvae. These data suggest that production of a highly specific metabolite in root exudates subdues detrimental nematodes, but stimulates beneficial ones, and illustrates a complex coevolution of plant compounds and various organisms in the rhizosphere.

The final research paper by James Ryalls and colleagues (2015) describes experiments that aim to replicate the true ecological complexity of plant–insect interactions within the context of global climate change and multiple biotic and abiotic stresses. Their study demonstrates the need to undertake experimental research to understand potential implications of future climate change and illustrates the complexity of interaction of multiple factors, suggesting that modelling is still a long way from being able to predict accurately the impacts of future climate change. The authors show that elevated CO_2_ concentration causes increases in both the foliar amino acid concentration of *Medicago sativa* and its capacity to support aphid colonization. However, these effects were eliminated at elevated temperatures. Further complexity was introduced when plants were subjected to simulated root herbivory.

Taken together, the papers in the current special issue highlight the quality and diversity of research currently ongoing in the fascinating field of plant–insect interactions. We hope that the reader finds the volume as enlightening as we did. Finally we would like to thank all of the contributors as well as the production team at the *Journal of Experimental Botany* who have been so helpful in the production of this work.
